# WNK1 in Malignant Behaviors: A Potential Target for Cancer?

**DOI:** 10.3389/fcell.2022.935318

**Published:** 2022-06-22

**Authors:** Ji-Ung Jung, Ankita B. Jaykumar, Melanie H. Cobb

**Affiliations:** Department of Pharmacology, UT Southwestern Medical Center, Dallas, TX, United States

**Keywords:** WNK1, OSR1, SPAK, TGF-β, EMT

## Abstract

Metastasis is the major cause of mortality in cancer patients. Analyses of mouse models and patient data have implicated the protein kinase WNK1 as one of a handful of genes uniquely linked to a subset of invasive cancers. WNK1 signaling pathways are widely implicated in the regulation of ion co-transporters and in controlling cell responses to osmotic stress. In this review we will discuss its actions in tumor malignancy in human cancers and present evidence for its function in invasion, migration, angiogenesis and mesenchymal transition.

## Introduction

WNK (With-No-Lysine) kinases were originally identified in a screen for novel members of the MAP kinase kinase (MAP2K) family (Xu et al., 2000; Verissimo and Jordan, 2001). The four WNK family members are characterized by an atypical placement of the catalytic lysine. These serine-threonine kinases participate in several functionally distinct signaling cascades controlling cellular responses to osmotic stress (Xu et al., 2005b; Kahle et al., 2005; Lenertz et al., 2005; Moriguchi et al., 2005; Anselmo et al., 2006; Gagnon et al., 2006; Vitari et al., 2006; Zagorska et al., 2007; Richardson et al., 2008; Roy et al., 2015). Many putative WNK substrates have been identified; the best-known are the related kinases OSR1 (oxidative stress responsive kinase1) and SPAK (STE20/SPS1-related proline/alanine-rich kinase) (Chen et al., 2004; Moriguchi et al., 2005; Vitari et al., 2005; Anselmo et al., 2006; Cope et al., 2006; Vitari et al., 2006; Richardson and Alessi, 2008). OSR1 and SPAK regulate ion co-transporters and will be discussed further below (Moriguchi et al., 2005; Vitari et al., 2005; Anselmo et al., 2006; Roy et al., 2015). In recent years, there is growing evidence that WNK1 is a critical kinase involved in various types of cancer (Chen et al., 2017; Gallolu Kankanamalage et al., 2018; Shahi Thakuri et al., 2020), but the exact mechanisms by which WNK1 modulates tumor progression are not well understood. Here we summarize the structure and turnover of WNK1 protein and evidence for its actions in tumor malignancy in human cancers.

### Overview of With-No-Lysine kinases Structure and Expression

In humans, the four WNK kinases, WNK1-4, range in size from 2833 amino acids (the longest isoform of WNK1) to 1243 amino acids (WNK4) (Verissimo and Jordan, 2001; Wilson et al., 2001; Delaloy et al., 2003; Vidal-Petiot et al., 2012). The four WNK family members share high homology in their kinase domains with 85–90% sequence identity, two autoinhibitory-like domains, a coiled-coil domain, and proline-rich (PXXP) motifs for protein-protein interactions (Xu et al., 2000; Verissimo and Jordan, 2001; Wilson et al., 2001; Xu et al., 2005a; Huang et al., 2007; Wang et al., 2008). Crystal structures of the kinase domains and autoinhibitory domains of multiple WNKs have been determined and some structures with small molecules bound are available (Min et al., 2004; Xu et al., 2005a; Moon et al., 2013). Outside of these regions, WNKs show low sequence identity, which is assumed to confer functional diversity for each of the WNK family members. Further, the abundance of low complexity sequence outside of the kinase domain makes structure determination challenging. Alphafold2 did not provide much greater clarity on the balance of the WNK1 molecule, for example, (Jumper et al., 2021; Varadi et al., 2021).

WNK1 and WNK4 have been extensively studied because they are responsible for pseudohypoaldosteronism type II (PHA II), a genetic disease characterized by hyperkalemic hypertension (Wilson et al., 2001; Susa et al., 2014). In contrast to other members of the WNK family which display a more tissue-restricted pattern of expression, WNK1 is widely and highly expressed in most animal tissues and cell types (Xu et al., 2000; Wilson et al., 2001; O'reilly et al., 2003; Vitari et al., 2005). The sequence of WNK1 is conserved across species (human WNK1 > 85% identical with mouse, pig 86%, rat 76%, and bovine 68%) and is recognizable in plants and many unicellular eukaryotes. The kinase activity of WNK1 is regulated *via* autophosphorylation on activation loop residues Ser382 and Ser378 (Xu et al., 2002). Details of mechanisms controlling autophosphorylation remain scant; however, importantly, chloride ion binding in the active site prevents autophosphorylation (Piala et al., 2014). This finding is relevant to WNK function as central regulators of ion homeostasis.

### With-No-Lysine kinases1 Turnover and Ion Homeostasis

Due to its importance for cellular homeostasis (Shekarabi et al., 2017), the abundance of WNK1 protein is tightly controlled by several layers of negative regulation (Mccormick and Ellison, 2011; Li et al., 2014; Roy et al., 2015; Dbouk et al., 2016). The set point in the total amount of WNK1 protein in a cell has a substantial impact on control of its downstream targets. All WNK family members share the highly conserved acidic degron motif near their autoinhibitory domain as a signal for destruction, which plays a critical role in the ubiquitin-dependent proteolytic process (Schumacher et al., 2014; Chen et al., 2022). The initial finding showed that the adaptor protein kelch-like family member 3 (KLHL3)-cullin3 (CUL3) E3 ligase complex is associated with WNK1 and WNK4 (Shibata et al., 2013). KLHL3 as a substrate adaptor binds to the conserved degron motif just C-terminal to the kinase domain in these WNKs to facilitate the recruitment of the CUL3 E3 ubiquitin ligase, thereby promoting ubiquitination and degradation (Shibata et al., 2013; Schumacher et al., 2014). Perturbations in this interaction have great effects on WNK-mediated electrolyte homeostasis, which was demonstrated by the finding that mutations in KLHL3-CUL3 also cause PHA II (Ohta et al., 2013; Mccormick et al., 2014; Lin et al., 2019).

Other ubiquitination pathways have also been connected to WNK1 turnover. The HECT-type ubiquitin ligase NEDD4-2 (also called NEDD4L) can bind to PY motifs in WNK1 for facilitating its ubiquitin-dependent proteasomal degradation (Heise et al., 2010; Roy et al., 2015). This is supported by the finding that WNK1 abundance was increased and OSR1/SPAK activation were observed in mice lacking NEDD4-2 (Roy et al., 2015; Al-Qusairi et al., 2017). We recently showed that WNK1 is degraded not only by the ubiquitin-proteasome pathway, but also by the lysosomal pathway (Jung et al., 2022). Non-lysosomal cysteine proteases calpain and caspase-3 were also able to influence WNK1 abundance. This study identified UBR5 (ubiquitin protein ligase E3 component N-recognin 5) as a previously unknown regulator of WNK1 turnover that mediates lysosomal degradation of WNK1 protein (Jung et al., 2022). Though UBR5 inhibition only modestly elevated WNK1 protein, that change caused a significant increase in phosphorylation of OSR1/SPAK, indicating that even small changes in WNK1 protein will have a significant impact on cellular processes. It is therefore likely that multiple degradative pathways in cells participate in the modulation of cellular WNK1 protein amount. WNK1 itself can also act as an adaptor for endosomal trafficking. WNK1 is thought to be crucial for glucose transporter GLUT1 and GLUT4 endosomal trafficking through regulating the Rab GTPase-activating protein AS160 (Akt substrate of 160 kDa) (Mendes et al., 2010; Tan et al., 2012; Kim et al., 2018; Henriques et al., 2020). WNK1 interacts with the endocytic scaffold protein intersectin which is involved in clathrin-mediated endocytosis that impacts recycling of ROMK (renal outer medullary potassium channel) (He et al., 2007; Wang et al., 2008). Moreover, we previously found that WNK1 negatively regulates autophagic degradation pathways through inhibition of class III phosphatidylinositol 3-kinase (PI3KC3) (Gallolu Kankanamalage et al., 2016). These results imply that WNK1 protein is degraded by multiple proteolytic pathways, whereas it is also a critical modulator of endocytic degradation. The mechanisms underlying the feedback between WNK1 turnover and its function as a trafficking regulator remain to be determined.

### With-No-Lysine kinases1, Angiogenesis, and Cell Junction Regulation

Phenotypes identified in mouse knockout studies demonstrated that WNK1 is required for angiogenesis. Angiogenesis is a highly regulated process that is turned on transiently during development, reproduction, and wound repair, which involves formation of new capillaries through sprouting or by splitting off from the original vessel (intussusception) (Folkman and Shing, 1992; Otrock et al., 2007). Homozygous disruption of the WNK1 gene results in a lethal developmental failure in mice around embryonic day E12, due to impaired angiogenesis (Xie et al., 2009; Xie et al., 2009). The phenotype of the WNK1 global knockout mouse mimics the defects caused by endothelial-specific ablation of WNK1 and is rescued either by endothelial-specific expression of WNK1 (Xie et al., 2009) or an activated form of OSR1 (Xie et al., 2013). Additionally, depletion of WNK1 and OSR1 decreased *in vitro* vascular cord formation and cell migration in endothelial cells (Dbouk et al., 2014; Jaykumar et al., 2022), indicating a crucial role of WNK1-activated OSR1 signaling in angiogenesis, and vascular remodeling.

Although a detailed mechanistic understanding of the effects of WNK1 on angiogenesis is lacking, decreased expression of WNK1 in cultured endothelial cells caused reduced expression of a number of factors that promote angiogenesis including Slug (SNAI2), vascular endothelial growth factor A (VEGF-A), and matrix metallo-proteinases (MMPs) (Dbouk et al., 2014). During vein graft remodeling, Slug mediates endothelial-mesenchymal transition (also called Endo-MT) *via* SMAD2/3-mediated transforming growth factor-β (TGF-β) signaling (Cooley et al., 2014; Welch-Reardon et al., 2014; Mahmoud et al., 2017), a widely known inducer of this transition (Nakajima et al., 2000; Pardali et al., 2017). We previously showed that WNK1 interacts with, and phosphorylates SMAD2 and regulates its function (Lee et al., 2007; Li et al., 2020). WNK1 signaling takes part in the regulation of TGF-β/SMAD-dependent Endo-MT for promoting onset of cell sprouting, migration and vascular remodeling. These and other findings indicate an effect of the WNK1 cascade on induction of a mesenchymal phenotype essential for endothelial wound healing (Yoshimatsu and Watabe, 2011; Welch-Reardon et al., 2014).

Partial Endo-MT is characterized by transient loss of an endothelial phenotype and acquisition of mesenchymal characteristics such as loss of cell-cell junctions, polarity and gain of motility to promote angiogenic sprouting and cell migration (Otrock et al., 2007; Welch-Reardon et al., 2015). TGF-β initiates cytoskeletal turnover and a drastic down-regulation and disintegration of tight junctions to promote migration in endothelial cells. Upon TGF-β stimulation, the TGF-β receptor type II redistributes into tight junctions which leads to their dissolution (Barrios-Rodiles, 2005; Ozdamar et al., 2005). Because OSR1 is a mediator of WNK1 action and because OSR1 was found to be a component of the TGF-β interactome (Barrios-Rodiles, 2005), we explored the possibility that WNK1 regulates OSR1 to influence endothelial tight junction turnover. We found that OSR1 is involved in WNK1-mediated regulation of turnover of tight junctions and adherens junctions, in part, through its interaction with occludin *via* a TGF-β-sensitive process. While occludin is not necessary for the formation of tight junctions, occludin is vital to tight junction integrity (Rao, 2009; Cummins, 2011). Interestingly, occludin is also important for directional migration of epithelial cells (Du et al., 2010). Control of both tight junction integrity and directional migration of endothelial cells are central to angiogenesis (Liu et al., 2016). One mechanism underlying the importance of WNK1/OSR1 to angiogenesis is the capacity to target occludin turnover. This same capacity may also contribute to effects of the WNK1 pathway on endothelial-mesenchymal transition. Furthermore, recent studies have shown that occludin is also involved in endothelial neovascularization and angiogenesis (Liu et al., 2016). In addition to effects on occludin, we identified other events that underlie WNK1-mediated control of angiogenesis including stabilization of TGF-β-regulated components AXL (a TAM receptor tyrosine kinase), ALK1 (a TGF-β receptor), SMAD2/3, RhoA, and VE-cadherin (Jaykumar et al., 2022).

Adherens junctions are required for endothelial cell stabilization and homeostasis because they promote contact inhibition of growth and decrease cell responsiveness to apoptotic stimuli. Adherens junctions in endothelial cells primarily consist of VE-cadherin (Dejana, 2004; Rudini et al., 2008). VE-cadherin is also known to be an endothelial cell-specific regulator of TGF-β/SMAD signaling (Rudini et al., 2008). TGF-β induces TGF-βRII association with VE-cadherin and this clustering promotes TGF-β signaling, which, in turn, destabilizes cell-cell junctions (Ma et al., 2020). Moreover, VE-cadherin is essential for TGF-β-induced endothelial cell migration (Rudini et al., 2008). VE-cadherin is a positive regulator of TGF-β-induced SMAD2/3 phosphorylation. TGF-β stimulation induces association of VE-cadherin with TGF-βRII/TGF-βRI and therefore it participates in maximal activation of the TGF-β pathway (Rudini et al., 2008). OSR1 was also shown to phosphorylate the SMAD2/3 linker region to promote TGF-β signaling (Li et al., 2020). We found that WNK1 inhibition leads to decreased localization of VE-cadherin at cell-cell junctions only in the presence of TGF-β, indicating that WNK1 actions are context dependent (Jaykumar et al., 2022).

### With-No-Lysine kinases1 and Cancer Prognosis

For the last two decades, the majority of studies on WNK1 have focused on their roles in hypertension and kidney function. Although multiple studies revealed that WNK1 is involved in major cancer-related signaling pathways such as PI3K-AKT, TGF-β and NF-κB (Xu et al., 2005c; Jiang et al., 2005; Lee et al., 2007; Yan et al., 2008), little is known regarding how WNK1 contributes to cancer progression. Recently, there has been growing interest in involvement of WNK1 in cancers. In fact, large-scale cancer database analysis from the cBioPortal for Cancer Genomics revealed that a high level of WNK1 expression has been observed in various tumor types including prostate, ovarian, testis and breast cancers (The cBioPortal for Cancer Genomics; http://cbioportal.org). As noted above, upregulation of WNK1 protein can result in increased activation of its downstream pathways and potential cancer-promoting actions. Utilizing transposon-mediated insertional mutagenesis for identifying candidate BC driver genes, WNK1 was identified as one of a handful of driver genes in high-risk invasive breast cancer (Chen et al., 2017).

**FIGURE 1:**
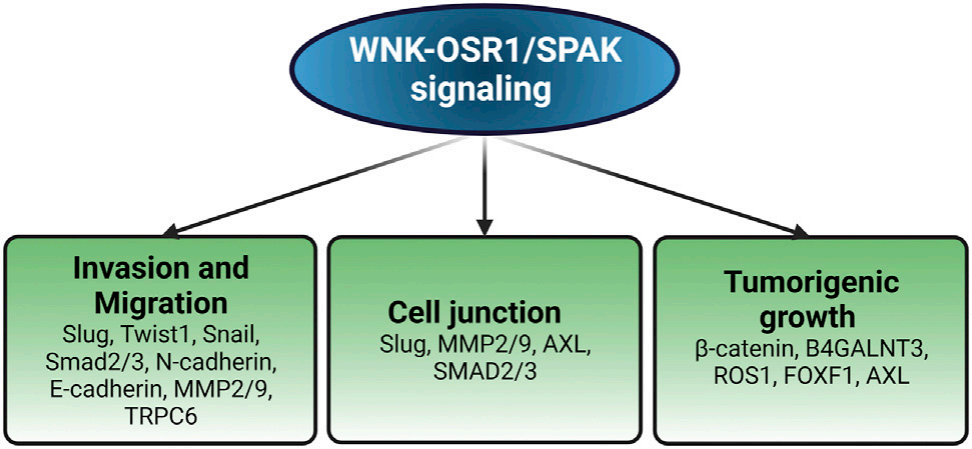
Pathological impacts of WNK1 in cancer. WNK1 regulates the expression of various molecules involved in different aspects of oncogenesis. Created with BioRender.com.

Paralleling findings in endothelial cells, WNK1 has been implicated in migration with epithelial-mesenchymal (EMT) features through knockdown studies in multiple cancer types, and also as a contributor to stem-like properties in metastatic breast cancers (Shyamasundar et al., 2016; Hung et al., 2017; Pio et al., 2017). Mechanistic information has suggested links to expression of EMT factors Slug and Snail, microRNA networks, changes in expression of cell surface proteins, altered vesicle trafficking, and effects on actin polymerization (Shyamasundar et al., 2016; Hung et al., 2017; Tanaka and Siemann, 2019). One study showed that WNK protects the interaction between β-Catenin and the glucose-induced degradation deficient (GID) complex, which includes an E3 ubiquitin ligase targeting β-Catenin, and that WNK regulates β-Catenin levels. Furthermore, WNK inhibitors induced β-Catenin degradation and suppressed xenograft tumor development in mice (Sato et al., 2020). Elevated WNK1 mRNA and protein have been detected in hepatocellular carcinoma (HCC) and significantly correlated with poor prognosis, highlighting the clinical significance of WNK1 in HCC progression (Ho et al., 2020). Knockdown and inhibition of WNK1 also decreased tumor-induced ectopic vessel formation and inhibited tumor proliferation in two zebrafish models transplanted with intestinal and hepatocellular carcinomas (Sie et al., 2020). Endothelial-specific overexpression of WNK1 enhanced tumorigenesis in transgenic carcinogenic zebrafish, supporting endothelial cell-autonomous effect of WNK1 in tumor production (Sie et al., 2020). In addition, WNK1 fusion to B4GALNT3 is a potential oncogenic driver in a patient with papillary thyroid carcinoma, which is the most common type of thyroid cancer (Costa et al., 2015). Another newly identified gene fusion WNK1-ROS1 (c-Ros oncogene-1) has been described as a novel driver of lung adenocarcinoma (Liu et al., 2019). WNK1 has been shown to promote tumorigenesis in cancers through its ability to promote cell proliferation *via* anti-apoptotic and pro-survival functions (Tu et al., 2011; Fulford et al., 2016; Wang et al., 2017). Importantly, the WNK1 signaling axis contributes to pathologic features of malignancies such as enhanced invasion, migration, adhesion and tumorigenicity, as shown in Figure 1. Thus, interfering with WNK1 expression may have therapeutic value in human cancers. In the following sections, we discuss different mechanisms by which WNK1 has been reported to support tumor malignancy.

### With-No-Lysine kinases1 and Epithelial–Mesenchymal Transition

The major characteristic of malignant tumors is a diffuse invasion into adjacent normal tissue and migration to a significant distance from the primary tumor area, a fundamental step in metastasis (Van Zijl et al., 2011; Clark and Vignjevic, 2015; Lambert et al., 2017). This characteristic is one of the most difficult challenges to target therapeutically in human cancers. Therefore, a clear understanding of the cellular and molecular mechanisms underlying the malignant behavior is crucial for the development of specific therapeutic strategies.

Multiple studies have implicated WNKs in cell migration in wound healing assays (Adams et al., 2017; Desjardins et al., 2019; Kim et al., 2019; Liu et al., 2020). A direct relationship between WNK1 and cell invasion in three-dimensional assays was described in human umbilical vein endothelial cells (HUVECs) (Dbouk et al., 2014). In that study, sprouting and invasion into the surrounding matrix were significantly diminished by WNK1 knockdown in spheroid sprouting assays. The impaired invasive phenotype of WNK1-depleted cancer cells was linked to downregulation of the EMT-associated transcription factor Slug which was partially rescued by overexpressing the WNK1-regulated kinases OSR1 or SPAK (Jaykumar et al., 2021). Consistent with this, it has recently been proposed that OSR1 depletion in MDA-MB-231 cells displays a decrease in expression of EMT transcription factors Twist1, Snail, and Slug at mRNA and protein levels, resulting in reduced invasion of breast cancer cells *in vitro* and *in vivo* (Li et al., 2021). As expected, OSR1 overexpression elevated levels of EMT transcription factors (Li et al., 2021). The pathological importance of complete or partial EMT has become clear from numerous studies and the molecular mechanisms governing malignant phenotypes of cancer cells such as invasion and metastasis formation are becoming clearer (Dang et al., 2015; Brabletz et al., 2018; Ramesh et al., 2020; Ribatti et al., 2020). Inhibition of WNK signaling with WNK463, an allosteric inhibitor of WNK kinase activity (Yamada et al., 2016; Zhang et al., 2016), significantly decreased expression of the EMT-related factor N-cadherin in breast cancer cells (Jaykumar et al., 2021). This mesenchymal marker is often highly expressed in cancers and has been shown to be associated with poor prognosis (Mariotti et al., 2007; Mrozik et al., 2018). Depletion of WNK1 in MDA-MB-231 cells significantly attenuated invasive potential in three-dimensional collagen matrices, and a similar result was also observed in cells treated with WNK463 (Jaykumar et al., 2021). In another study in MDA-MB-231 cells, miRNA-93 was reported to suppress WNK1 expression, resulting in a decrease in the invasiveness of the cells through upregulation of epithelial markers CLDN1, CLDN3 and CDH1 (Shyamasundar et al., 2016). Additionally, it was shown that overexpression of WNK1 in hepatocellular carcinoma enhanced the expression of MMP-2 and MMP-9, which are EMT-related proteolytic enzymes that can promote degradation of the extracellular matrix, EMT, and invasion (Dong et al., 2020). Thus, diverse studies place WNK1 as a regulator of EMT during the invasion of different types of cancer cells.

Evidence generated from several studies suggests that WNK1 is a key in cancer cell migration *via* regulation of EMT. Since the initial finding through *in vitro* scratch wound healing assays that knockdown of WNK1 reduced migration of mouse neural progenitor cell line C17.2 accompanied by morphological changes (Sun et al., 2006), numerous studies have identified WNK1 regulation of the migratory phenotype in cancer cells. In non-small cell lung cancer cell lines, WNK1 silencing leads to decreased EMT molecules N-cadherin and Snail, and increased E-cadherin, resulting in reduced migration of CL1-5 and H1299 cells (Hung et al., 2017). Also, work from our lab has shown that WNK1 inhibition in five different breast cancer cell lines MDA-MB-231, BT-549, BT-20, HCC1569, and HCC1419 significantly reduced the migratory capacity concomitantly with the reduction in N-cadherin protein expression (Jaykumar et al., 2021).

### With-No-Lysine kinases, Ion Cotransporters and Migration

As discussed above, the WNK-OSR1/SPAK signaling pathway is key in the regulation of electrolyte homeostasis through regulating ion co-transporters of the SCL12A family. These include the Na^+^K^+^2Cl^−^ co-transporters 1 and 2 (NKCC1 and NKCC2), the Na^+^Cl^−^ co-transporter (NCC), and multiple K^+^Cl^−^ co-transporters (KCC) that modulate the flux of ions across the cell membrane (Gamba, 2005; Flatman, 2008; Richardson and Alessi, 2008). WNK1 has also been demonstrated to regulate cell surface expression of ion channels including the epithelial Na^+^ channel (ENaC) and renal outer medullary K^+^ channel (ROMK, Kir 1.1) (Xu et al., 2005b; Cope et al., 2006; Lazrak et al., 2006). We recently showed that Kir2.1 and Kir2.3 channels harbor an alternative SPAK/OSR1 binding motifs (RxFxV), leading to their plasma membrane localization (Taylor et al., 2018). Alterations of electrolyte homeostasis are frequently observed in cancer patients (Miltiadous et al., 2008; Rosner and Dalkin, 2014; Li et al., 2020; Bennet et al., 2021).

These ion transporters and channels have frequently been implicated in the regulation of migration and invasion (Cuddapah and Sontheimer, 2011; Schwab and Stock, 2014; Stock and Schwab, 2015; Martial, 2016). WNK1 knockdown in primary glioma cells results in attenuated phosphorylation of NKCC1, the ubiquitously expressed ion co-transporter, in response to the chemotherapeutic drug temozolomide, reducing glioma cell migration (Garzon-Muvdi et al., 2012). In a further study, Köchl et al. described the mechanistic connection between WNK1 and NKCC1 on migration of T cells *in vitro* and *in vivo*. In that study, WNK1 was shown to negatively regulate integrin-mediated adhesion through inhibition of the small GTPase RAP1, and positively regulate migration through promoting OSR1/SPAK-mediated NKCC1 activation, proposed to account for cell volume changes required for cell migration (Kochl et al., 2016). The importance of NKCC1 in cancer cell invasion and migration is supported by studies carried out in numerous systems. In glioblastoma cells, NKCC1 silencing impedes invasion and migration, accompanied by inhibition of MMP-2 and MMP-9 (Sun et al., 2020). The invasive ability of hepatocellular carcinoma was regulated by NKCC1 (Zhou et al., 2017). We found that depletion of OSR1 also reduced migration of breast cancer cells; however, inhibition of NKCC1 was not as effective as OSR1 depletion in slowing migration (Jaykumar et al., 2021). Finally, NKCC1 was reported to facilitate EMT of human gastric cancer cells through the MAPK-JNK signaling pathway, resulting in enhanced invasion and migration (Wang et al., 2021). WNK1 regulation of calcium homeostasis has been previously described (Lee et al., 2004; Xu et al., 2005a; Liu et al., 2015). A recent study has hinted that actions of WNK1 on calcium homeostasis may participate in cancer progression. WNK1 affects TRPC6 (canonical transient receptor potential channel)-mediated Ca2^+^ influx, leading to migration in clear-cell renal-cell carcinoma (Kim et al., 2019). These results highlight the functional importance of ion homeostasis regulated by WNK1 in cancer cell invasion and migration.

### With-No-Lysine kinases1 and AXL

Phenotypic plasticity in several cancers is dependent on the expression of the receptor tyrosine kinase AXL. In normal mammary epithelial cells, AXL is a driver of stemness (Fujino et al., 2017; Engelsen et al., 2020). Interestingly, several characteristics of epithelial-mesenchymal transition overlap with those in endothelial cells (Welch-Reardon et al., 2015). Endothelial-expressed AXL is known to modulate angiogenesis (Gallicchio et al., 2005; Sacha et al., 2005; Li et al., 2009). The catalytic activity of AXL induces endothelial tube formation *in vitro* and knockdown of AXL in breast cancer cells and in endothelial cell co-culture impairs this process (Sacha et al., 2005; Li et al., 2009). AXL was identified as a downstream effector of TGF-β and modulates expression of TGF-β/SMAD-dependent target genes involved in cell migration in hepatocellular carcinoma (Bauer et al., 2012; Lee et al., 2014; Reichl et al., 2015). Moreover, inhibition of AXL decreases autocrine TGF-β signaling in hepatocellular carcinoma and impairs secretion of pro-angiogenic factors in breast cancer cells which in turn affects the function of endothelial cells in co-culture and *in vivo* (Reichl et al., 2015; Tanaka and Siemann, 2019). Paracrine angiogenic factors have also been shown to be expressed in endothelial cells, suggestive of an autocrine signaling loop (Lee et al., 2007). In view of these observations, we investigated the potential of an AXL and WNK1 signaling collaboration in endothelial cells to regulate endothelial cell migration and tube formation. We found that inhibiting WNK1 decreased expression of the tyrosine kinase AXL, apparently not due to a change in AXL mRNA (Jaykumar et al., 2021).

**FIGURE 2:**
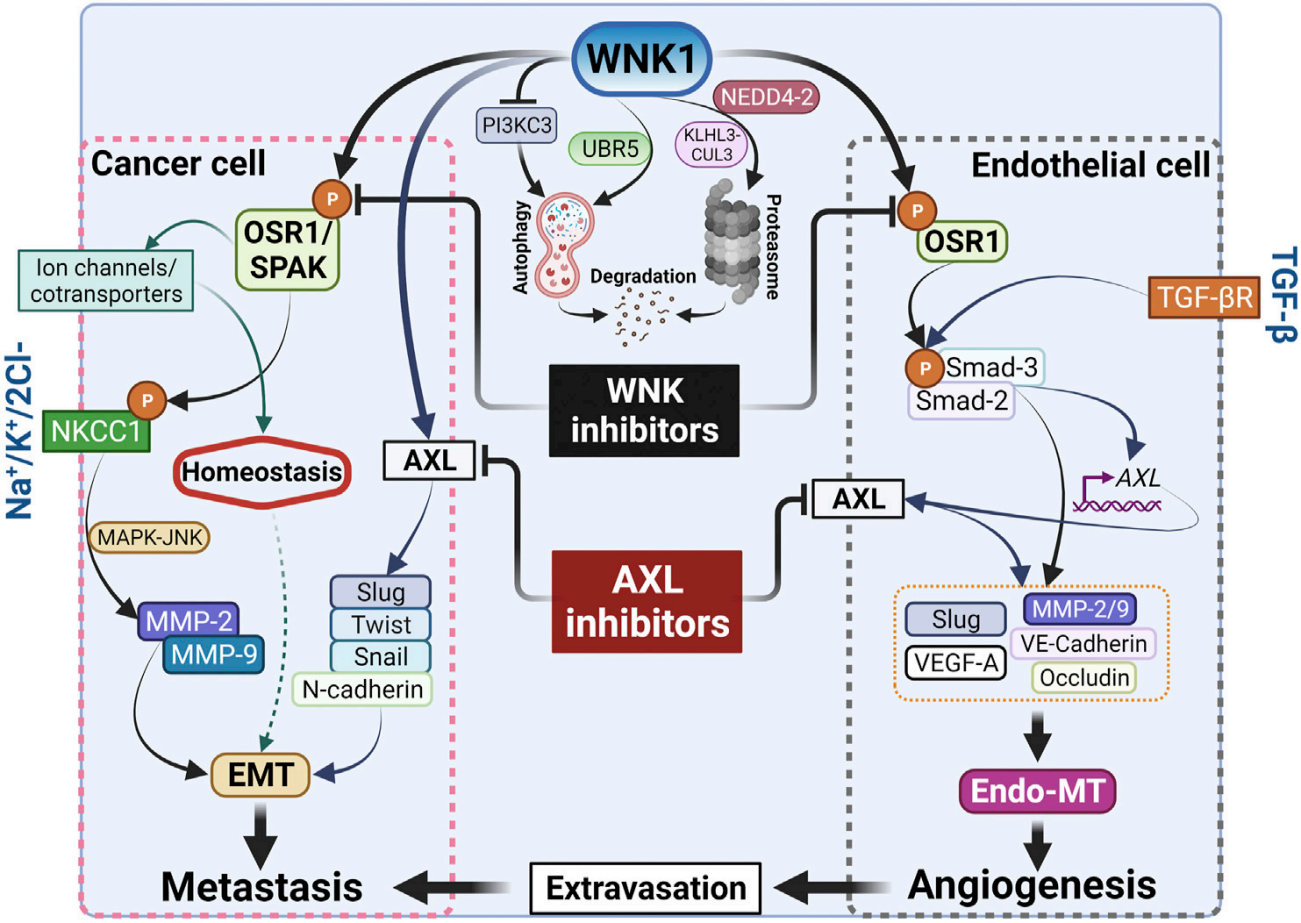
Cancer metastasis and angiogenesis by WNK1. WNK1 is a key in the metastasis cascade *via* regulation of EMT-effectors and -transcription factors. Created with BioRender.com.

AXL expression is associated with metastasis and poor prognosis in a variety of tumor types including breast cancer (Oh et al., 2011; Dang et al., 2015; Garrido-Castro et al., 2019). BGB324 is a first-in-class AXL inhibitor, currently in phase II clinical trials, exhibiting promising therapeutic characteristics. It displays AXL-selective antitumor and antimetastatic activity in murine models of breast cancer (Garrido-Castro et al., 2019). AXL inhibition in tumor cells decreases the secretion of pro-angiogenic factors such as endothelin and VEGF-A and impairs functional properties of endothelial cells *in vivo*, suggesting its important role in the initiation of tumor angiogenesis (Tanaka and Siemann, 2019). In our recent study, we found elevated phospho-OSR1 in bone metastatic cells, suggesting that increased WNK activity may be a feature of breast cancers that can metastasize to multiple sites. We showed that inhibition of WNK1 reduces tumor volume and dispersion of metastatic cells in a mouse xenograft model of metastatic breast cancer, in part, *via* a network involving Slug and AXL (Jaykumar et al., 2021). Interestingly, we previously reported that mRNAs encoding these factors were also decreased upon knockdown of WNK1 in endothelial cells (Dbouk et al., 2014). Perhaps WNK1 knockdown diminishes these mRNAs in part *via* suppression of AXL (Dang et al., 2015). The connection revealed here between AXL and WNK1 raises the possibility that WNK1 may be a therapeutic option in other AXL-dependent tumor types as well. We also found that inhibition of WNK1 *via* WNK463, had a more pronounced effect than the AXL inhibitor to attenuate migration in MDA-MB-231 cells. Yet, the combination of AXL and WNK inhibitors was more effective at reducing migration than inhibition of either alone (Jaykumar et al., 2021). This observation warrants future studies examining the combination of AXL and WNK inhibitors on tumor progression and metastasis in animal models, which could potentially inform future clinical trials (Figure 2).

## Conclusion

In summary, we briefly highlight four points that may be considered in future analyses of the actions of WNK1 and other WNK family members in cancers. It should be clear that WNK1 has related actions in angiogenesis and cancer. How many of these related actions are shared by other WNK family members is not clear. The control WNK1 exerts over ion transport is a significant factor in its actions in cancers but may be overshadowed by its potential impact on cell phenotype. In both endothelial cells and cancer cells, WNK1 has the capacity to induce migration and enable transition towards a mesenchymal phenotype (Figure 2). This occurs in certain contexts, e.g., in endothelial cells during wound repair, but what other triggers exist and what contexts determine when these actions of WNK1 will overtake its homeostatic functions are not established or yet recognized. We close with the question why and when does WNK1 switch from being a homeostatic housekeeper to instead promoting EMT.
